# Hypoglycemic Effects of Extracts Obtained from Endemic *Betonica bulgarica* Degen and Neič

**DOI:** 10.3390/plants13101406

**Published:** 2024-05-18

**Authors:** Iva Slavova, Tea Genisheva, Gabriela Angelova, Vasilyan Chalumov, Teodora Tomova, Mariana Argirova

**Affiliations:** Department of Chemical Sciences, Faculty of Pharmacy, Medical University of Plovdiv, 15A Vassil Aprilov Str., Plovdiv 4002, Bulgariateodora.tomova@mu-plovdiv.bg (T.T.); mariyana.argirova@mu-plovdiv.bg (M.A.)

**Keywords:** endemic species, *Betonica bulgarica*, postprandial glucose, acarbose, synergism, phytochemicals

## Abstract

The increasing prevalence of diabetes mellitus, together with the limited access of many patients to conventional antidiabetic drugs and the side effects resulting from their use, are the reason for the ever-increasing need for new agents. One of the most important strategies used in the therapy of this disease is to reduce the postprandial blood glucose level by inhibiting the carbohydrate-degrading enzymes α-amylase and α-glucosidase. The purpose of the present study was to provide in vitro evidence for the potential hypoglycemic effect of leaf and inflorescence aqueous extracts of Bulgarian endemic species *Betonica bulgarica* Degen and Neič. Total phenolic and flavonoid contents and antioxidant activities were determined by spectrophotometric methods. Qualitative and quantitative determinations of principal phenolic acids and flavonoids were performed using HPLC with a dual absorbance detector. The plant extracts were able to retard the enzymatic breakdown of starch to glucose with 50% inhibiting concentrations of 1.86 mg/mL and 1.54 mg/mL respectively for leaf and flower extract. Some of the plant constituents are proven inhibitors of α-amylase and/or α-glucosidase, but their adsorption on starch seems to be one additional mechanism for the inhibition of glucose release. Combination index analysis carried out with binary mixtures of acarbose and plant extracts showed a tendency toward synergism with an increase in concentrations and level of inhibition.

## 1. Introduction

Traditional medicine has been a valuable tool for the prevention or adjuvant treatment of causes or symptoms of several diseases and pathological conditions. Natural plant sources are assumed to be of low toxicity and affordable, especially in low-income countries [1]. Another reason for the popularity of natural-based remedies is their accessibility and the relatively light regime of registration with national government regulatory offices. Regulations concerning phytopharmaceuticals in both raw forms or processed and standardized pharmaceutical formulations are not so strict, although they vary from country to country. In Bulgaria, such plant-derived formulations are classified as food additives [2]. 

Diabetes mellitus is a serious metabolic disorder caused by the abnormality of carbohydrate metabolism, which is linked to low blood insulin levels or the insensitivity of target organs to insulin. Nearly 800 medicinal plants from different geographic regions are reported to have antidiabetic activity according to ethnobotanical studies [3]. Bulgarian traditional medicine recommends tens of herbs to assist diabetes management; their effects are most often related to a reduction in blood glucose or stimulation of insulin secretion [4]. Several metabolic pathways involved in the development of diabetes have been proposed as targets of medicinal plants; among them are the inhibition of starch-hydrolyzing enzymes α-amylase and α-amyloglucosidase [5], inhibition of dipeptidyl peptidase-IV [6], activation of peroxisome proliferator-activated receptors-γ (PPAR-γ) [7], reduction in osmotic stress through inhibition of aldose reductase [8], or combination of several mechanisms [9]. Apart from these possible mechanisms, diabetes is accompanied by oxidative stress and an increase in reactive oxygen species that can have a deleterious effect on many cells, tissues, and organs [10]. Plants are rich in different natural antioxidants, in particular tannins, flavonoids, and vitamins C and E that can maintain β-cells performance and temper or retard some diabetes complications.

One of the most effective strategies used in controlling diabetes is to reduce the postprandial glucose level in the blood by the inhibition of carbohydrate-digesting enzymes α-amylase and α-glucosidase. Both salivary and pancreatic α-amylase cleave α-1,4-glycosidic bonds in amylase and amylopectin to oligosaccharides (dextrins) and maltose. Alpha-amyloglucosidase, which is an integral part of intestinal absorptive cell membranes and some enzymes in the brush border (maltase, isomaltase, sucrase, and lactase) continue the process of oligo- and disaccharide hydrolysis to monosaccharides, mainly glucose [11]. Acarbose, a microbial pseudo-oligosaccharide, isolated from strains of *Actinoplanes* sp. (in the order *Actinomycetales*), is known to inhibit the intestinal α-glucosidases, α-amylase, sucrase, and maltase [12]. This leads to decreased release of glucose from starch-rich foods and dose-related delay in or reduction in the postprandial increase in blood glucose and triglycerides. Acarbose is used in clinical practice under different trade names and its addition to existing treatment with metformin or sulfonylurea is associated with improvements in life expectancy and quality-adjusted life expectancy and provides excellent value for money over patient lifetimes [13]. 

**Figure 1:**
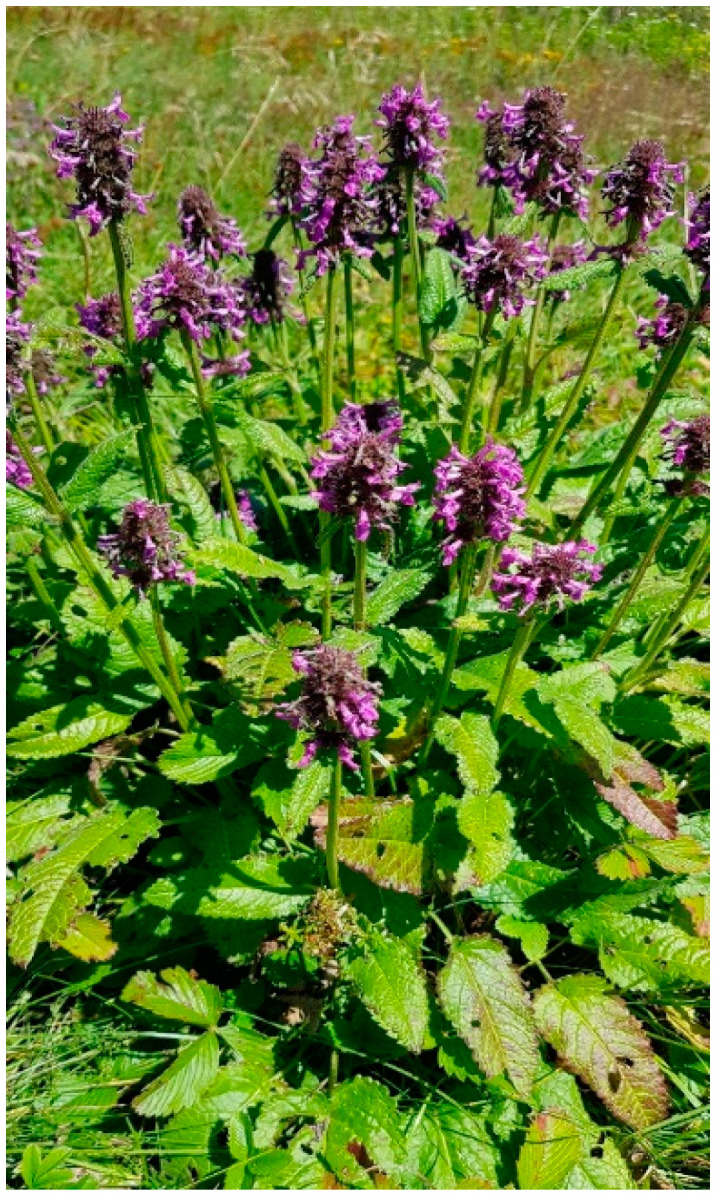
Aerial part of *Betonica bulgarica* Degen and Neič (Bulgarian Betony).

*Betonica bulgarica* Degen and Neič (Bulgarian Betony) from the family Lamiaceae (Figure 1) is a Bulgarian endemic species protected under the Biological Diversity Act (2002) and included in the *Red Book of Bulgaria, Vol.1—Plants and Fungi* under the category “endangered” [14]. The species was first described by the Hungarian botanist Árpád von Degen and Bulgarian Ivan Neychev in 1906 [15].

*Betonica bulgarica* is close to *Betonica officinalis* L. (Stachys Betony), and is used as a medicinal plant. Infusions of *Betonica officinalis* dried leaves have been used in Bulgaria, Serbia, Egypt, and Montenegro to treat skin disorders, for antibacterial purposes, against headache, nervous tension, anxiety, and menopausal problems [16,17]. Herb extract exhibited an inhibitory effect on α-amylase with a 50% inhibiting concentration of 4.20 mg/mL [18]. To the best of our knowledge there have been no clinical human trials supporting the use of *Betonica officinalis* for any of the above-mentioned indications.

Few research groups have studied the value of *Betonica bulgarica* as a plant with therapeutic potential. The antimicrobial potential of methanolic extracts obtained from leaves, flowers, seeds, stems, and roots has been recently reported [19]. All extracts demonstrated either low and statistically insignificant activity against *E. coli* or a lack of such but root extracts of *Betonica bulgarica* exhibited moderate antibacterial activity against *S. aureus* and *B. cereus.* Extract of aerial parts of the plant in 70% methanol exerted in vitro cytotoxic effects against two tumor cell lines and demonstrated immunomodulatory activity [20]. The composition of essential oil isolated from *Betonica bulgarica* has also been analyzed [21], but there are no data on their use in traditional medicine.

This study was undertaken in search of properties of *Betonica bulgarica* extracts that are beneficial for human health, namely the possibility of their use as a hypoglycemic agent. Two types of solvents were used to obtain leaf and flower extracts from *Betonica bulgarica*. Aqueous infusions are easily prepared at home, allowing many bioactive compounds, including some volatile components, to be extracted from plants and taken by humans in a water-soluble form. On the other hand, some bioactive ingredients are poorly soluble or insoluble in water, for example terpenes, phytosterols, and vitamins A and E, but have relatively suitable solubility in methanol [22]. Dry methanolic extracts are more suitable for the production of commercial formulations because of their prolonged stability. In this study, we compared the properties of both aqueous and methanolic extracts in terms of their phytochemical composition and antioxidant properties. Furthermore, we tested the abilities of *Betonica bulgarica* aqueous extracts to retard the enzymatic breakdown of starch to glucose and investigated some possible mechanisms underlying this effect.

## 2. Results

### 2.1. Phytochemical Screening and Antioxidant Properties of Aqueous and Methanolic Extracts

Phenolic compounds are the most abundant secondary metabolites of plants and are ubiquitous in all plant organs. The total phenolic content and flavonoid content of aqueous and methanolic extracts obtained from *Betonica bulgarica* leaves and inflorescences were quantified using standard phytochemical methods. Two different methods, scavenging of 2,2-diphenyl-1-picrylhydrazyl free radical (DPPH) and measurement of total reducing capacity were applied for comparison of their antioxidant activity (Table 1).

### 2.2. Chromatographic Profile and Chemical Constituents

Dual wavelength monitoring of effluent after chromatographic separation of the extracts allowed the identification of some of their principal constituents (Table 2).

### 2.3. Hypoglycemic Effect of Aqueous Extracts Obtained from Betonica bulgarica

**Figure 2:**
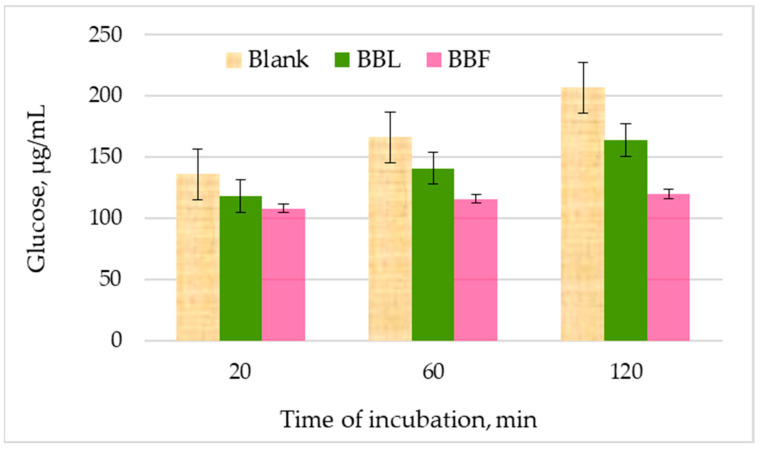
Time course of enzymatic hydrolysis of starch by a mixture of α-amylase and α-amyloglucosidase (blank) and under the same conditions but in the presence of 2 mg/mL aqueous plant extracts from leaves (BBL) and flowers (BBF) of *Betonica bulgarica* Degen and Neič.

In this study, we used as a substrate a sample with known content of digestible starch and a mixture of pancreatic α-amylase and α-glucosidase whose concentrations were optimized by the manufacturer according to the procedure of Englyst et al. [23]. Glucose, the product of the enzymatic reaction was specifically quantified using the standard peroxidase-coupled glucose method (GOD-POD). The level of glucose released from starch in the presence of hydrolyzing enzymes was followed over 2 h (Figure 2) since the time for food digestion in the small intestine, where pancreatic α-amylase and α-glucosidase act, is 2–4 h [23]. 

### 2.4. Studies on Adsorption of Plant Constituents onto Starch 

**Figure 3:**
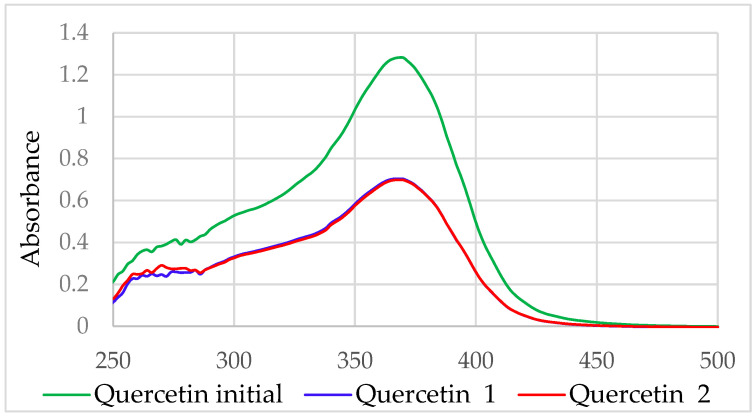
UV-VIS spectra of quercetin (0.025 mg/mL) in 50 mM maleate buffer pH 6.0 before (quercetin initial line) and after incubation with starch (quercetin 1 and 2 lines, two parallel samples).

We investigated the ability of starch to retain compounds from the three major groups of polyphenols: flavonoids (glycosylated and aglycon), hydroxy derivatives of benzoic acid and cinnamic acid, some of which were also present in *Betonica bulgarica* extracts. There was no observed change in the spectra of caffeic, ferulic, gallic, and 4-oxybenzoic acid, as well as in the spectrum of rutin before and after incubation with starch, showing the lack of adsorption of these compounds on its surface. However, the intensity of the absorption maximum of quercetin at 368 nm after incubation with starch was only 55% of that of the initial solution (Figure 3), indicating retention of the dissolved flavonoid on the polysaccharide surface.

**Figure 4:**
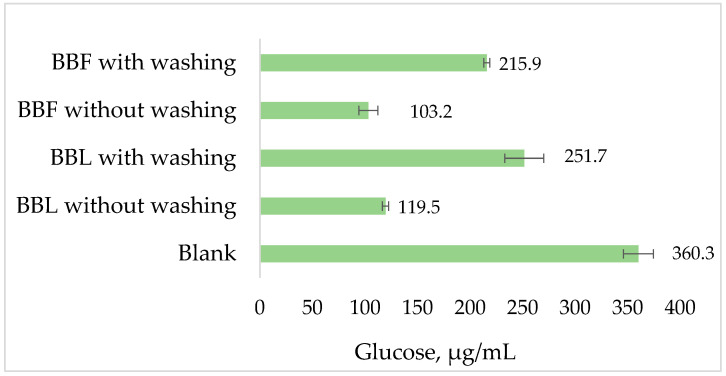
Glucose concentration after enzymatic hydrolysis of starch in the absence (blank) and the presence of *Betonica bulgarica* extracts: from leaves (BBL) and flowers (BBF).

When the starch-retained components of the extracts were removed by washing with methanol, the concentration of glucose released from the starch was significantly higher compared to the unwashed samples, but still lower than that released from the starch in the absence of extract (Figure 4). 

### 2.5. Study of Combined Effects of Acarbose and Plant Extracts

Concentration-effect studies of the aqueous extract from *Betonica bulgarica* in the concentration range 1–15 mg/mL for a 2 h incubation period showed that the 50% reduction (IC_50_) in the amount of glucose released from starch in the presence of α-amylase and α-amyloglucosidase is caused by 1.86 ± 0.12 mg/mL of the leaf extract and 1.54 ± 0.10 mg/mL of the flower extract (see Supplementary Material 1). 

**Figure 5:**
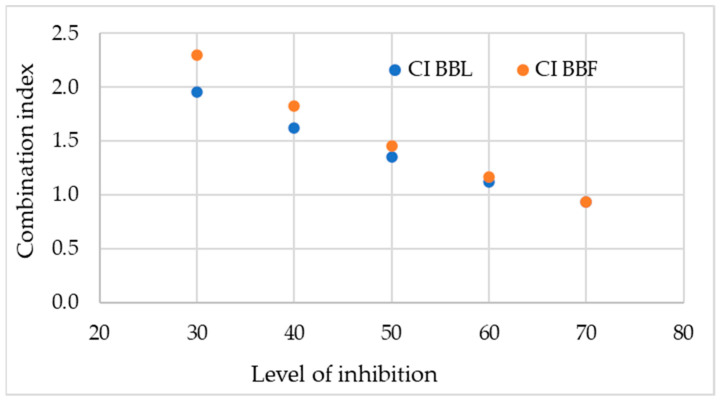
Evaluated type of interaction based on calculated combination index between acarbose and leaf (BBL) or flower (BBF) extracts obtained from *Betonica bulgarica*.

Five different concentration levels of acarbose and plant extracts, providing 30, 40, 50, 60, and 70% inhibition of glucose release when applied independently, were used to quantitively evaluate the type of interactions in their binary mixtures (Figure 5). The combination index (CI) was calculated according to the equation proposed by Chou and Talalay [24]. When CI < 1, synergism is indicated; CI = 1 is indicative of an addition effect, and if CI > 1, antagonism is indicated. 

## 3. Discussion

The idea to investigate the inhibitory effect of *Betonica bulgarica* extracts toward the enzymes involved in starch hydrolysis arose after the elucidation of their chemical composition. Caffeic and chlorogenic acids were shown to be α-amylase inhibitors with concentrations causing 50% enzyme inhibition (IC_50_) of 3.68 μg/mL and 9.10 μg/mL respectively [25]. The same compounds also inhibited α-glucosidase (IC_50_ 4.98 μg/mL for caffeic and 9.24 μg/mL for chlorogenic acid). Salicylic acid has also been mentioned as an antidiabetic agent [26,27]. Inhibition of α-amylase is mainly due to the capacity of polyphenols to bind on active site of enzyme through hydrogen bonds between the hydroxyl groups of the polyphenol ligands and the catalytic center of the enzyme, together with hydrophobic interactions between the aromatic rings of phenolic compounds and tryptophan residues of α-amylase [28,29].

Quercetin, one of the most abundant flavonoid pigments, improves oral glucose tolerance, as well as pancreatic β-cell function to secrete insulin. It inhibits α-glucosidase and DPP-IV enzymes, which prolongs the half-life of glucagon-like peptide-1 (GLP-1) and glucose-dependent insulinotropic polypeptide (GIP) [30]. Quercetin is also known as a suitable antioxidant, but its therapeutic application is limited by its very sparing solubility in water, around 10^−4^ mol/L at 298 K, according to Abraham et al. [31]. Molecular docking has been used to identify the flavonoids capable of binding in the active center of α-amylase [32]. 

Phytochemical screening revealed that the total phenolic content of *Betonica bulgarica* aqueous and methanolic extracts was not significantly different when comparing different solvents and between leaves and flowers. The quantity of the flavonoids was also similar. Methanolic extracts, however, demonstrated better radical scavenging properties and total reducing capacity than the corresponding aqueous solutions, indicating either a larger amount of extractable antioxidants in methanol or the presence of compounds that are non-extractable in water but soluble in methanol. Our further experiments were focused on the properties of the extracts in water that are typically used in traditional medicine.

Further chromatographic analysis quantified some of the phenolic compounds in the extracts. Rutin, quercetin and hispidulin, the aglycone of hesperidin, were previously found in *Betonica bulgarica* extracts by Tzanova et al. [33]. The presence of chlorogenic and caffeic acid in leaves and flowers in amounts commensurable with those found by us was also reported [20]. Among phenolic acids, vanillic and salicylic acid were also presented in significant amounts. Despite this, the identified compounds exhibited only 4.45% of the dry matter of the leaf extract and 5.17% of that of the flower extract. Maybe some of the other constituents do not absorb at the wavelengths used (280 and 370 nm), for example, carbohydrates and organic acids, and part of the dry matter, are inorganic. Some other glycosylated phenolic compounds were qualified in methanolic extracts from the aerial part of *Betonica bulgarica*, but they were probably in minor concentrations in our extracts and below the limit of detection [34].

Research on the effects of natural sources on α-amylase activity most often uses the assay originally proposed by Miller [35]. It is based on the reduction in 3,5-dinitrosalicylic acid by the aldehyde group of the glucose released from starch after enzymatic hydrolysis. The method is fast, robust, and applicable in most laboratories, but it also has some drawbacks, the major one being that plant extracts contain a significant number of compounds that, like glucose, have reducing properties. This was also confirmed by our analysis of total reducing capacity demonstrated by *Betonica bulgarica* extracts (Table 1). Therefore, the parallel running of a sizable number of blanks without enzyme and/or substrate is required. The colorimetric reagent we used in our studies specifically measures the amount of glucose released after enzymatic hydrolysis. In addition, to obtain a homogeneous reaction mixture, the Miller’s method uses soluble starch. It is partially hydrolyzed starch to dextrins, which is far from the real starch state in foods [36]. Polyphenols can be adsorbed onto the surface of granular starch, resulting in a change in its microstructure and this can affect its digestion [37,38]. The ability of flavonoids to form complexes with starch depends on their structure, and it was found that with an increase in number of hydroxyl groups in their skeleton, their inhibitory activity increases [39].

Time course of starch breakdown to glucose in the presence or absence of extracts was followed over 2 h. The results show that already in the first 20 minutes the release of glucose from the starch is slowed down in the presence of the plant extracts, and after 2 hours the amount of released glucose is nearly 50% lower in the presence of the *Betonica bulgarica* extracts, especially the flower extract (Figure 2). 

At the end of the concentration-effect experiments, the coloration of residual non-hydrolyzed starch was clearly visible. After washing the pellet twice with methanol, the UV-VIS spectra of these methanol solutions were scanned. All of them showed intense absorption between 324 and 332 nm, the typical region of flavonoids absorption. This led us to hypothesize that binding of polyphenolic compounds to starch may lead to reduced substrate digestion. To test this hypothesis, we conducted experiments to assess the probability of interaction between starch and some phenolic compounds. 

Among the polyphenol compounds tested by us, only quercetin demonstrated significant adsorption on starch but not its glycosylated analog rutin. The difference in the behavior of the two flavonoids in terms of their adsorption can be attributed to the glycosylation of rutin and thus, to influence their ability to retard glucose hydrolysis. This hypothesis is also confirmed by Fernandes et al. [40] who reported that glycosylation of the hydroxyl group of flavonoids at position C-3 of the chromane ring can have a negative effect on their inhibitory activity. 

Based on our results shown in Figure 4 we can conclude that the formation of adsorption complexes between the flavonoid and starch is the main reason for the delay of substrate hydrolysis, but the inhibition of α-amylase and/or α-amyloglucosidase enzyme activity by some of the extracts constituents could also play a role in the total effect.

Both tested extracts showed a weaker inhibiting effect than acarbose, whose IC_50_ values have been reported to be 5 ± 0.1 μM for α-amylase and 328 ± 7 μM for α-glucosidase [41]. Although acarbose is generally considered safe, it was recently included in the FDA data set as a compound causing drug-induced liver injury [42]. One of the possible approaches to reduce the therapeutic dose of acarbose and thus limit its adverse side effects is to combine it with bioactive compounds of natural origin [43]. The tested combinations of acarbose and *Betonica bugarica* extracts showed that at lower concentrations of acarbose and plant extracts CI was above 1 but there was a clear tendency to additive and synergistic effect demonstrated by higher concentrations and inhibition level 70% (Figure 5). Further characterization of the interaction between acarbose and plant extracts in terms of optimal concentrations of both components may be an essential step in the development of safer combinations and offer new therapeutic strategies to control hyperglycemia in diabetic patients. 

Of course, one can argue that whatever health-promoting effects the extracts of *Betonica bulgarica* have, it is a protected species and cannot and should not be harvested uncontrolled and in large quantities. However, in recent years, many efforts have been made to find the optimal agrotechnical conditions allowing this botanical species not only to be conserved, but also cultivated while preserving its uniqueness [44,45].

## 4. Materials and Methods

### 4.1. Plant Material

Leaves and inflorescences (flowers) from *Betonica bulgarica* (Lamiaceae) (Figure 1) were collected during flowering in June–July from the Bulgarka Nature Park, the area of Uzana, Stara Planina Mountain. Plant material was identified by Prof. Plamen Stoyanov (Department of Botany and Methods of Biology Teaching, Faculty of Biology, University of Plovdiv “Paisii Hilendarski”). The collected raw materials were dried in shadow at room temperature and powdered. A voucher specimen for *Betonica bulgarica* (n. 062646) was deposited at the Herbarium of the Agricultural University, Plovdiv, Bulgaria.

### 4.2. Preparation of Aqueous and Methanol Extracts

Water extracts were prepared according to the recommendations of traditional medicine. Typically, a ratio of 1:10 dried plant/boiling deionized water (18.2 mΩ/cm^2^) was used for the preparation of infusions. The same ratio was used for ultrasound-assisted triple extraction with methanol. The combined methanolic extracts were further concentrated under vacuum at 30 °C to 1/3 of the initial volume and then filtered. The dry matter of the extracts was determined gravimetrically.

### 4.3. Extract Analyses

#### 4.3.1. Determination of Total Phenolic Content

Spectrophotometric measurements were taken in a Spectronic Camspec M550 (Spectronic Camspec Ltd., Leeds, UK) instrument. Total phenolics were determined using classical Folin and Ciocalteu colorimetric method [46] and expressed in terms of mg gallic acid equivalents (GAE), which were determined from standard plot of gallic acid (y = 0.1004x + 0.0104, R^2^ = 0.9999x). 

#### 4.3.2. Determination of Total Flavonoid Content

Determination of total flavonoid content was carried out by aluminum chloride assay using quercetin for calibration plots (y = 0.0374x − 0.0282, R^2^ = 0.9872); the results were presented as quercetin equivalents (QE) in mg [47]. All the results referred to 1 g dry matter in the extract.

#### 4.3.3. Free Radical Scavenging Activity

The method based on 2,2-diphenyl-2-picrylhydrazyl (DPPH) discoloration in the presence of antioxidants was used for the determination of the free radical scavenging ability of the extracts. The experimental conditions of the assay were set according to those used by Paun et al. [18]. The antiradical activity was defined as the dry extract in μg providing 50% inhibition (IC_50_) of the initial DPPH and was calculated from a graph plotting absorbance at 517 nm against extract concentration.

#### 4.3.4. Ferric-ferrozine Assay of Total Antioxidant Capacity

The total antioxidant capacity of plant extracts was measured according to the method proposed by Berker et al. [48]. Solutions of water-soluble vitamin E analog Trolox (6-hydroxy-2,5,7,8-tetramethylchroman-2-carboxylic acid) in 70% methanol in the concentration range 50–250 μM were used for the calibration plot and the results for antioxidant capacity were expressed as Trolox equivalents, i.e., µmol Trolox causing the same reduction in the ferric-ferrozine complex as 1 g dry extract. 

#### 4.3.5. Qualitative and Quantitative Analysis of Extracts by High Performance Liquid Chromatography

Qualitative and quantitative determinations of phenolic acids and flavonoids were performed using Waters 1525 Binary Pump HPLC systems (Waters, Milford, MA, USA), equipped with Waters 2484 dual Absorbance Detector (Waters, Milford, MA, USA) and Supelco Discovery HS C18 column (5 µm, 25 cm × 4.6 mm), operated under the control of Breeze 3.30 software. The sample was injected at 20 µL volume and gradient elution at a flow rate of 1.0 mL/min was performed by using the gradient program described previously by Sherova et al. [49]. The chromatograms were acquired at 280 nm for gallic acid, protocatehuic acid, (+)-catechin, vanillic acid, syringic acid, (−)-epicatechin, p-coumaric acid, salicylic acid, and hesperidin whereas chlorogenic acid, caffeic acid, ferulic acid, rutin, rosmarinic acid, quercetin and kaempferol were detected at 360 nm. For the identification of phenolic acids and flavonoids their retention times were compared with those of reference compounds; the latter were also used for calibration plots and quantification of the analytes using Breeze 3.30 software (see Supplementary Materials 2 and 3).

#### 4.3.6. Enzymatic Hydrolysis of Starch in the Presence of *Betonica bulgarica* Extracts

The in vitro hypoglycemic property of aqueous extracts was evaluated using a digestible starch and resistant starch assay kit (K-DSTRS) from Megazyme (Bray, Ireland) according to the manufacturer’s protocol and rescaled for the purpose of the assay. Briefly, 25 mg starch provided by the manufacturer was suspended in 910 µL 50 mM maleate buffer (pH 6.0 containing 2 mM CaCl_2_) for the control samples or aliquot of concentrated plant extract needed to obtain the desired concentration and made up to 910 µL with maleate buffer. All samples were vortexed, and a 90 µL solution of pancreatic α-amylase and α-amyloglucosidase (20 mg/mL) was added. Samples were incubated for 2 h at 37 °C with occasional inversion of the tubes. The enzymatic reaction was stopped by adding 100 µL supernatant to 900 µL 50 mM AcOH. The concentration of glucose released from starch was determined by the reagent and glucose standard solution provided by the manufacturer using the peroxidase-coupled glucose method GOD-POD [50].

#### 4.3.7. Inhibition of Glucose Release in the Presence of *Betonica bulgarica* Extracts

To prove the adsorption of some low molecular mass bioactive constituents of plant extracts on the starch surface, solutions in maleate buffer in concentrations that provide a maximum UV absorption of around 1–1.5 units (0.025 mg/mL for quercetin and rutin, 0.05 mg/mL for caffeic and ferulic acid, 1 mg/mL for 4-oxybenzoic acid and 0.5 mg/mL for gallic acid) were added to 25 mg starch, vigorously vortexed, and incubated for 30 min at 37 °C. UV-VIS spectra of these solutions were run before and after incubation with starch.

Two parallel sets of experiments were set to study the effect of adsorption on the amount of enzymatically released glucose in the presence of plant extracts. One of them was as described above but the other set was initially incubated for 30 min with plant extract. After brief centrifugation, the supernatant was carefully removed, and the insoluble starch was washed with MeOH (2 × 1 mL) and quickly dried under vacuum. Then, the separated supernatants were returned to the tubes, and the enzymatic reaction was started by adding enzyme solution. After 2 h the reaction was stopped, and the glucose concentration was determined. The two methanol washing solutions were combined, and their UV-VIS spectra were recorded.

Inhibition of glucose release was calculated by means of equation:

$$I=\left(1-\frac{Glc\;sample}{Glc\;blank}\right)\times 100$$

where *I* is the inhibition level in %, Glc _sample_ is the glucose concentration in the presence of extract or binary mixture, and Glc _blank_ is the glucose concentration released in the absence of extract.

The combination index (CI) was calculated according to the following equation [24]:

$$CI=\left(\frac{Cacarbose1}{Cacarbose2}\right)+\left(\frac{Cextract1}{Cextract2}\right)$$

where C_acarbose2_ and C_extract2_ are the concentrations of acarbose and plant extract producing the same inhibition level when used individually, whereas C_acarbose1_ and C_extract1_ are the corresponding concentrations in binary mixture producing the same level of inhibition.

#### 4.3.8. Statistical Analysis

All analytical assays were carried out in triplicate and expressed as mean values ± standard error of the mean (SEM). Microsoft Excel 2010 (2004, Microsoft, Redmond, WA, USA) with Real Statistics Resource Pack installed was used to perform statistical processing and graphical layout of the experimental data obtained. Statistical differences were reported as significant at *p* < 0.05. 

## 5. Conclusions

Infusions of *Betonica bulgarica* Degen and Neič prepared according to the recommendations of traditional medicine are rich in health-beneficial compounds many of which are known as inhibitors of α-amylase. The ability of some of the extract constituents to adsorb onto starch and thus delay its enzymatic hydrolysis is an additional mechanism by which the postprandial glucose level may be reduced. The natural origin of plants, combined with the pleasant aroma of the infusions could be an important consideration for many patients of using their therapeutic potential.

## Figures and Tables

**Table 1:** Comparison between some phytochemical and antioxidant parameters of methanolic and aqueous extracts obtained from leaves (BBL) and flowers (BBF) of *Betonica bulgarica*.

Phytochemical Parameters	BBL/MeOH	BBL/H_2_O	BBF/MeOH	BBF/H_2_O
Total phenolic content (GAE/g)	118.0 ± 8.3	139.8 ± 11.8	124.8 ± 3.7	137.3 ± 9.2
Of which flavonoids (QE/g)	11.6 ± 0.1	19.3 ± 0.2 *	13.4 ± 0.7	20.5 ± 0.3 *
Antioxidant properties				
Radical-scavenging properties (DPPH method, IC_50_, μg/mL)	29.1 ± 0.8	74.2 ± 2.6 *	36.0 ± 1.0	46.2 ± 1.6 *
Total reducing capacity (Trolox equivalents, μmol)	991.7 ± 53.7	449.5 ± 16.2	814.8 ± 31.2	694.4 ± 12.1

Data are per gram dry matter of the extracts. Asterisk indicates statistically significant difference (*p* < 0.05) between aqueous and methanolic extract obtained from the same plant part.

**Table 2:** Content of some principal phenolic compounds in aqueous extracts of leaves (BBL) and flowers (BBF) of *Betonica bulgarica*.

Compound	Amount in BBL, mg/g	Amount in BBF, mg/g
Phenolic acids		
Gallic acid	N.A.	N.A.
Protocatechuic acid	0.35	0.57
Vanillic acid	1.81	0.94
Caffeic acid	0.41	0.26
Syringic acid	0.04	0.07
p-Coumaric acid	0.23	0.25
Chlorogenic acid	4.06	1.20
Ferulic acid	0.44	0.75
Salicylic acid	26.54	38.87
Rosmarinic acid	1.78	0.66
Flavonoids		
Rutin	1.92	1.15
Hesperidin	4.44	4.33
(+)-Catechin	0.67	1.05
(−)-Epicatechin	0.12	0.23
Quercetin	1.27	1.28
Kaempferol	0.46	0.12

Data refers to dry matter of the extract. N.A.: not available.

## Data Availability

The raw data supporting the conclusions of this article will be made available by the authors on request.

## Supplementary Materials

The following supporting information can be downloaded at: https://www.mdpi.com/article/10.3390/plants13101406/s1, Figure S1. Concentration-effect plots for: A. Leaf extract; B. Flower extract; C. Acarbose; D. Binary mixture leaf extract-acarbose; E. Binary mixture flower extract-acarbose. Since the concentration of acarbose is three orders of magnitude lower than that of extracts, only the concentration of extract is shown. Figure S2. Chromatographic profiles and retention times for principal constituents of *Betonica bulgarica* leaf extract. Figure S3. Chromatographic profiles and retention times for principal constituents of *Betonica bulgarica* flower extract.
