# Supplementary material for: Hypoglycemic Effects of Extracts Obtained from Endemic Betonica bulgarica Degen and Neič
Source: Plants (Basel). 2024 May 18;13(10):1406. doi: 10.3390/plants13101406 (PMC11125167; doi:10.3390/plants13101406)
Supplement: Supplementary file 1 [file plants-13-01406-s001.zip › Figure S1. Concentration-effect plots.pdf]

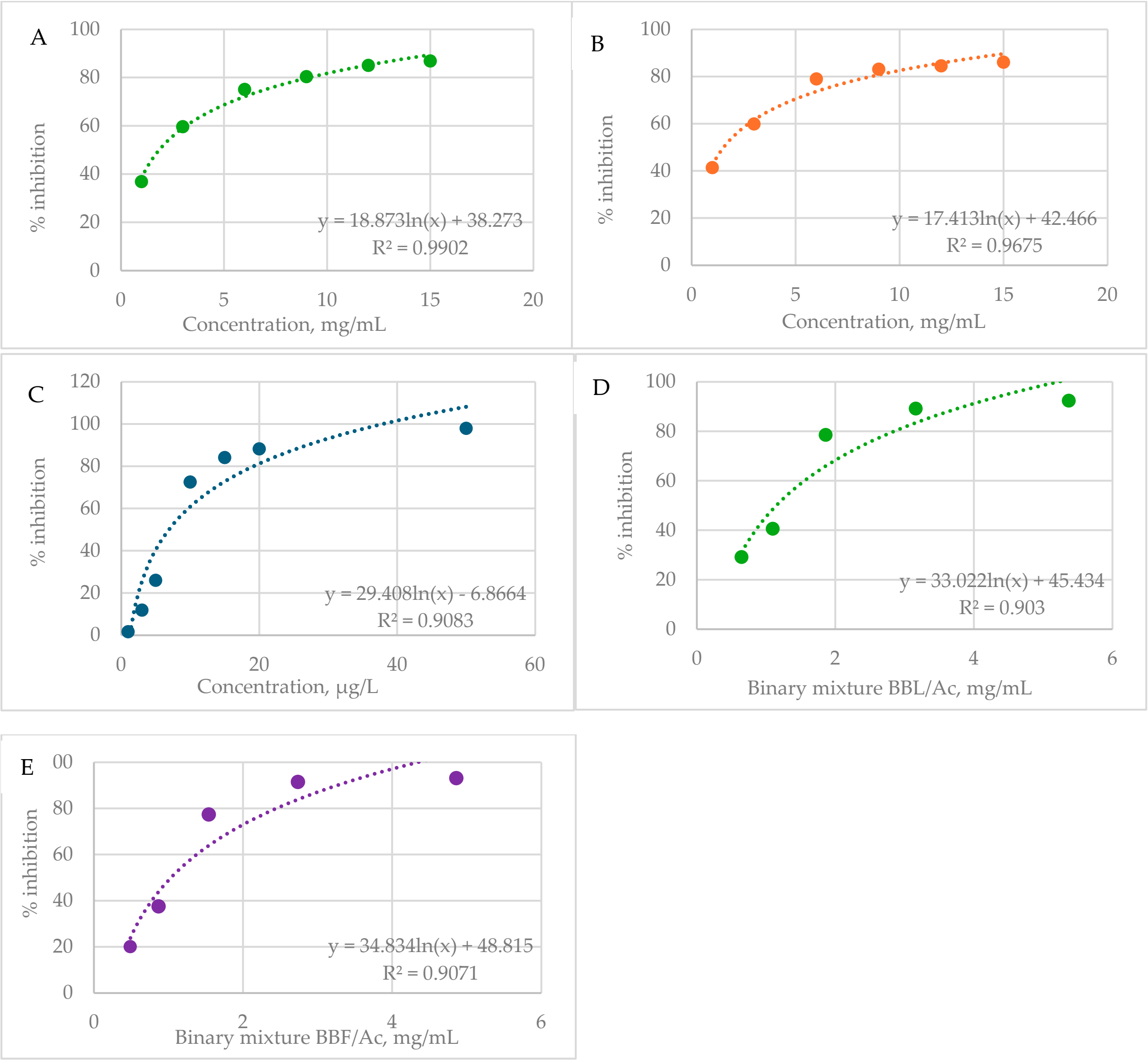

Figure S1. Concentration-effect plots for: A. Leaf extract; B. Flower extract; C. Acarbose; D. Binary mixture leaf extract-acarbose; E. Binary mixture flower extract-acarbose. Since the concentration of acarbose is three orders of magnitude lower than that of extracts, only the concentration of extract is shown.
