# Supplementary material for: Hypoglycemic Effects of Extracts Obtained from Endemic Betonica bulgarica Degen and Neič
Source: Plants (Basel). 2024 May 18;13(10):1406. doi: 10.3390/plants13101406 (PMC11125167; doi:10.3390/plants13101406)
Supplement: Supplementary file 1 [file plants-13-01406-s001.zip › Figure S2. BBL Chromatograms & RT.pdf]

SAMPLE INFORMATION

Sample Name:	BBL_1	Acquired By:	System
Sample Type:	Unknown	Sample Set Name:	
Vial:	999	Acq. Method:	PolyPhenols
Injection #:	3	Date Acquired:	02.9.2021 a. 11:57:12
Run Time:	75,00 Minutes	Injection Volume:	20,00 ul

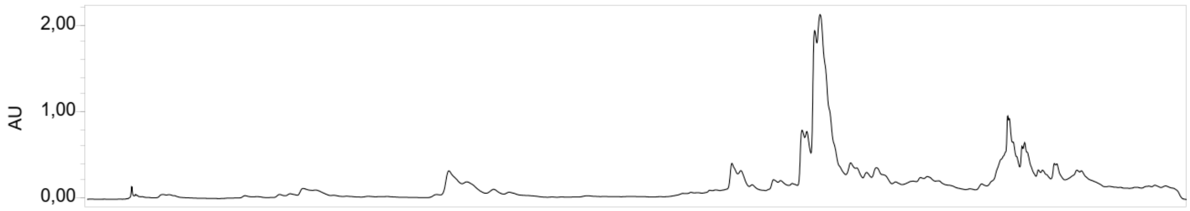

	Channel Description	Peak Name	RT (min)	Area (V*sec)	% Area	Height (V)	Amount	Units
1	Pa 280 nm		0,246	691	0,00	72		
2	Pa 280 nm		0,945	541	0,00	62		
3	Pa 280 nm		1,080	914	0,00	126		
4	Pa 280 nm		1,205	2040	0,00	154		
5	Pa 280 nm		2,217	1404	0,00	111		
6	Pa 280 nm		2,233	655	0,00	107		
7	Pa 280 nm		3,072	1315513	0,20	142756		
8	Pa 280 nm		3,334	419196	0,06	55347		
9	Pa 280 nm		3,436	592476	0,09	43534		
10	Pa 280 nm		3,778	570265	0,09	27400		
11	Pa 280 nm		4,196	346993	0,05	18526		
12	Pa 280 nm		4,578	178202	0,03	13943		
13	Pa 280 nm		5,198	1620019	0,25	51927		

	Channel Description	Peak Name	RT (min)	Area (V*sec)	% Area	Height (V)	Amount	Units
14	Pa 280 nm		5,603	3024633	0,47	49790		
15	Pa 280 nm	Gallic acid	6,405					
16	Pa 280 nm		7,900	170221	0,03	5666		
17	Pa 280 nm		8,700	35368	0,01	2386		
18	Pa 280 nm		9,699	194180	0,03	6040		
19	Pa 280 nm		10,792	1359525	0,21	32154		
20	Pa 280 nm	Protocatehuic acid	11,602	959969	0,15	20573	28,697	ug/ml
21	Pa 280 nm		12,560	226626	0,03	9765		
22	Pa 280 nm		13,149	1453590	0,22	44756		
23	Pa 280 nm		13,876	2139111	0,33	52065		
24	Pa 280 nm		14,757	5488846	0,85	112271		
25	Pa 280 nm		15,594	4935936	0,76	92669		
26	Pa 280 nm		16,840	1269245	0,20	31945		
27	Pa 280 nm		17,726	1116585	0,17	20441		
28	Pa 280 nm		19,195	819719	0,13	17106		
29	Pa 280 nm		19,993	368649	0,06	13397		
30	Pa 280 nm	(+)-Catechin	20,484	762012	0,12	14553	55,267	ug/ml
31	Pa 280 nm		21,567	96756	0,01	4696		
32	Pa 280 nm		22,237	46748	0,01	2158		
33	Pa 280 nm		23,853	1122278	0,17	36054		
34	Pa 280 nm	Chlorogenic acid	24,694	17013491	2,63	308828	411,563	ug/ml
35	Pa 280 nm		25,912	12213157	1,88	180511		
36	Pa 280 nm	Vanillic acid	27,744	4739709	0,73	93904	148,015	ug/ml
37	Pa 280 nm	Caffeic acid	28,807	4571680	0,71	59600	50,868	ug/ml
38	Pa 280 nm		31,739	67695	0,01	3381		
39	Pa 280 nm		32,379	67636	0,01	3226		
40	Pa 280 nm	Syringic acid	33,283	39265	0,01	1739	2,992	ug/ml
41	Pa 280 nm		34,131	611473	0,09	12630		
42	Pa 280 nm		35,266	45811	0,01	2128		
43	Pa 280 nm	(-)-Epicatechin	36,164	5393	0,00	479	9,971	ug/ml
44	Pa 280 nm		37,143	180455	0,03	4304		
45	Pa 280 nm		37,607	137291	0,02	3565		
46	Pa 280 nm		39,083	104216	0,02	3935		

	Channel Description	Peak Name	RT (min)	Area (V*sec)	% Area	Height (V)	Amount	Units
47	Pa 280 nm		40,651	1635559	0,25	42422		
48	Pa 280 nm		40,876	420265	0,06	42205		
49	Pa 280 nm		41,191	1487351	0,23	55365		
50	Pa 280 nm		41,674	1502162	0,23	51836		
51	Pa 280 nm	p-Coumaric acid	42,487	2681721	0,41	80484	18,978	ug/ml
52	Pa 280 nm		42,892	2507193	0,39	84132		
53	Pa 280 nm		43,995	14492505	2,24	393358		
54	Pa 280 nm		44,604	10883467	1,68	309210		
55	Pa 280 nm	Ferulic acid	45,368	7191816	1,11	146970	90,997	ug/ml
56	Pa 280 nm		46,833	7424794	1,15	205376		
57	Pa 280 nm		47,311	7576982	1,17	195989		
58	Pa 280 nm		48,103	5174020	0,80	163212		
59	Pa 280 nm		48,776	15654521	2,42	779646		
60	Pa 280 nm		49,099	17325051	2,67	763982		
61	Pa 280 nm	Salicylic acid	49,639	34979398	5,40	1930010	2176,415	ug/ml
62	Pa 280 nm		50,003	119179160	18,39	2113700		
63	Pa 280 nm		52,087	13933643	2,15	404596		
64	Pa 280 nm		52,499	8920704	1,38	345685		
65	Pa 280 nm	Rutin	53,174	9536446	1,47	294228	587,019	ug/ml
66	Pa 280 nm	Hesperidin	53,847	22247721	3,43	348721	363,990	ug/ml
67	Pa 280 nm		55,149	7066560	1,09	186536		
68	Pa 280 nm	Rosmarinic acid	56,345	10648983	1,64	196008	226,674	ug/ml
69	Pa 280 nm		56,850	6878679	1,06	238308		
70	Pa 280 nm		57,310	10772704	1,66	250535		
71	Pa 280 nm		58,080	18086512	2,79	200893		
72	Pa 280 nm		60,216	4336283	0,67	111349		
73	Pa 280 nm		61,029	6573630	1,01	168170		
74	Pa 280 nm		62,801	38286214	5,91	950982		
75	Pa 280 nm		62,933	11433298	1,76	902663		
76	Pa 280 nm	Quercetin	63,200	12096368	1,87	644339	388,646	ug/ml
77	Pa 280 nm		63,777	6539519	1,01	608760		
78	Pa 280 nm		63,948	23891847	3,69	646267		
79	Pa 280 nm		64,886	5525203	0,85	332190		

IM-BAS

Project Name: project
Reported by User: System

	Channel Description	Peak Name	RT (min)	Area (V*sec)	% Area	Height (V)	Amount	Units
80	Pa 280 nm		65,167	11933419	1,84	330148		
81	Pa 280 nm	Kaempherol	65,980	6564243	1,01	407623	195,610	ug/ml
82	Pa 280 nm		66,139	11261618	1,74	403292		
83	Pa 280 nm		67,512	16246542	2,51	331935		
84	Pa 280 nm		67,820	22577457	3,48	324870		
85	Pa 280 nm		69,467	1285908	0,20	143361		
86	Pa 280 nm		69,698	6609638	1,02	147368		
87	Pa 280 nm		70,466	3043896	0,47	136105		
88	Pa 280 nm		70,923	2783193	0,43	127531		
89	Pa 280 nm		71,469	3652867	0,56	136665		
90	Pa 280 nm		71,636	2928164	0,45	136754		
91	Pa 280 nm		72,419	6029732	0,93	151793		
92	Pa 280 nm		72,827	5574846	0,86	161700		
93	Pa 280 nm		73,558	10313371	1,59	154785		

SAMPLE INFORMATION

Sample Name:	BBL_1	Acquired By:	System
Sample Type:	Unknown	Sample Set Name:	
Vial:	999	Acq. Method:	PolyPhenols
Injection #:	3	Date Acquired:	02.9.2021 a. 11:57:12
Run Time:	75,00 Minutes	Injection Volume:	20,00 ul

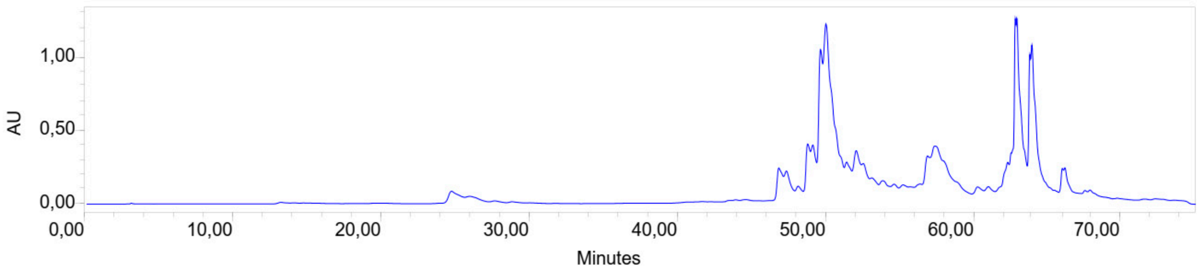

Channel: 2487Channel 2 Channel Desc.: FL 360 nm Processing Method: PolyPhenols UV

	Channel Description	Peak Name	RT (min)	Area (µV*sec)	% Area	Height (µV)	Amount	Units
1	FL 360 nm		3,052	89780	0,02	5451		
2	FL 360 nm		10,099	16054	0,00	481		
3	FL 360 nm		13,149	358155	0,10	9487		
4	FL 360 nm		14,029	143020	0,04	4349		
5	FL 360 nm		14,667	105571	0,03	3018		
6	FL 360 nm		17,577	10081	0,00	602		
7	FL 360 nm		18,060	12316	0,00	624		
8	FL 360 nm		19,424	47963	0,01	2648		
9	FL 360 nm		19,802	109381	0,03	2800		
10	FL 360 nm		20,318	104285	0,03	2610		
11	FL 360 nm		22,742	18613	0,01	539		
12	FL 360 nm	Chlorogenic acid	24,695	4702854	1,29	84968	332,799	ug/ml
13	FL 360 nm		25,906	3344014	0,92	50288		

	Channel Description	Peak Name	RT (min)	Area (V*sec)	% Area	Height (V)	Amount	Units
14	FL 360 nm		27,608	945306	0,26	18677		
15	FL 360 nm	Caffeic acid	28,789	655834	0,18	12759	33,352	ug/ml
16	FL 360 nm		29,988	299417	0,08	5473		
17	FL 360 nm		31,730	43100	0,01	1463		
18	FL 360 nm		33,883	11726	0,00	549		
19	FL 360 nm		36,279	23564	0,01	755		
20	FL 360 nm		36,769	37945	0,01	894		
21	FL 360 nm		38,075	13658	0,00	591		
22	FL 360 nm		40,600	319265	0,09	8186		
23	FL 360 nm		40,916	125186	0,03	8596		
24	FL 360 nm		41,615	621449	0,17	11794		
25	FL 360 nm		42,209	398707	0,11	11112		
26	FL 360 nm		42,795	223089	0,06	10389		
27	FL 360 nm		43,530	645758	0,18	20559		
28	FL 360 nm		43,903	706169	0,19	24333		
29	FL 360 nm		44,591	1454008	0,40	26029		
30	FL 360 nm	Ferulic acid	45,423	505061	0,14	18444	35,761	ug/ml
31	FL 360 nm		46,172	614984	0,17	19926		
32	FL 360 nm		46,817	6546047	1,80	242367		
33	FL 360 nm		47,325	7199671	1,97	221683		
34	FL 360 nm		48,117	3429663	0,94	118112		
35	FL 360 nm		48,772	8147565	2,23	406324		
36	FL 360 nm		49,112	9145475	2,51	399858		
37	FL 360 nm		49,640	18729665	5,14	1051367		
38	FL 360 nm		50,003	58352526	16,00	1225419		
39	FL 360 nm		51,394	7946436	2,18	283336		
40	FL 360 nm		52,045	11657228	3,20	361097		
41	FL 360 nm		52,521	7551863	2,07	273858		
42	FL 360 nm	Rutin	53,096	5864223	1,61	175801	157,304	ug/ml
43	FL 360 nm		53,839	6482788	1,78	156238		
44	FL 360 nm		54,608	4154267	1,14	132678		
45	FL 360 nm		55,206	4549033	1,25	128334		
46	FL 360 nm	Rosmarinic acid	55,679	2854811	0,78	115675	145,953	ug/ml

	Channel Description	Peak Name	RT (min)	Area (V*sec)	% Area	Height (V)	Amount	Units
47	FL 360 nm		56,353	3925003	1,08	134272		
48	FL 360 nm		56,869	7839095	2,15	326074		
49	FL 360 nm		57,358	36575747	10,03	393789		
50	FL 360 nm		60,251	4677697	1,28	115476		
51	FL 360 nm		60,977	4412089	1,21	117166		
52	FL 360 nm		62,284	9631167	2,64	283213		
53	FL 360 nm		62,526	3487810	0,96	349337		
54	FL 360 nm		62,808	12344401	3,39	1266189		
55	FL 360 nm		62,900	29319651	8,04	1265318		
56	FL 360 nm	Quercetin	63,792	8959832	2,46	1021250	104,347	ug/ml
57	FL 360 nm		63,921	32960673	9,04	1083608		
58	FL 360 nm		65,437	1837187	0,50	93476		
59	FL 360 nm	Kaempherol	65,983	3382917	0,93	243128	37,815	ug/ml
60	FL 360 nm		66,146	8585691	2,35	247105		
61	FL 360 nm		67,505	1935997	0,53	91852		
62	FL 360 nm		67,845	6036948	1,66	95096		
63	FL 360 nm		69,500	369001	0,10	37315		
64	FL 360 nm		69,686	2898875	0,80	40240		
65	FL 360 nm		71,593	1736798	0,48	33868		
66	FL 360 nm		72,272	1531479	0,42	36468		
67	FL 360 nm		72,774	1039704	0,29	33162		
68	FL 360 nm		73,560	1821941	0,50	27123		
