# Supplementary material for: Hypoglycemic Effects of Extracts Obtained from Endemic Betonica bulgarica Degen and Neič
Source: Plants (Basel). 2024 May 18;13(10):1406. doi: 10.3390/plants13101406 (PMC11125167; doi:10.3390/plants13101406)
Supplement: Supplementary file 1 [file plants-13-01406-s001.zip › Figure S3. BBF Chromatograms & RT.pdf]

IM-BAS

Project Name: project
Reported by User: System

SAMPLE INFORMATION

Sample Name:	BBF_1	Acquired By:	System
Sample Type:	Unknown	Sample Set Name:	
Vial:	999	Acq. Method:	PolyPhenols
Injection #:	4	Date Acquired:	02.9.2021 a. 13:13:13
Run Time:	75,00 Minutes	Injection Volume:	20,00 ul

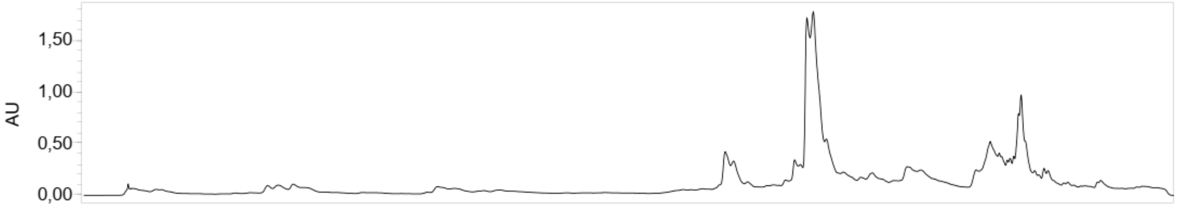

Channel: 2487Channel 1 Channel Desc.: Pa 280 nm Processing Method: PolyPhenols UV

	Channel Description	Peak Name	RT (min)	Area (µV*sec)	% Area	Height (µV)	Amount	Units
1	Pa 280 nm		1,006	1639	0,00	72		
2	Pa 280 nm		1,226	316	0,00	44		
3	Pa 280 nm		1,283	383	0,00	48		
4	Pa 280 nm		1,612	136	0,00	30		
5	Pa 280 nm		3,081	1540175	0,32	107634		
6	Pa 280 nm		3,304	520919	0,11	68989		
7	Pa 280 nm		3,423	3575993	0,74	66211		
8	Pa 280 nm		5,030	1938487	0,40	54887		
9	Pa 280 nm		5,372	3510619	0,73	52748		
10	Pa 280 nm	Gallic acid	6,405					
11	Pa 280 nm		7,927	757161	0,16	12144		
12	Pa 280 nm		10,447	1051929	0,22	15578		
13	Pa 280 nm	Protocatehuic acid	11,481	1094016	0,23	18742	32,802	ug/ml

	Channel Description	Peak Name	RT (min)	Area (V*sec)	% Area	Height (V)	Amount	Units
14	Pa 280 nm		12,672	2920536	0,61	85815		
15	Pa 280 nm		13,391	4566406	0,95	90359		
16	Pa 280 nm		14,432	4210334	0,87	98313		
17	Pa 280 nm		15,162	3848739	0,80	66323		
18	Pa 280 nm		16,549	838531	0,17	20766		
19	Pa 280 nm		17,375	995144	0,21	15777		
20	Pa 280 nm		19,213	532171	0,11	14051		
21	Pa 280 nm	(+)-Catechin	19,762	846630	0,18	12237	61,049	ug/ml
22	Pa 280 nm		21,853	23562	0,00	1045		
23	Pa 280 nm		23,001	3277	0,00	260		
24	Pa 280 nm		23,657	376358	0,08	15136		
25	Pa 280 nm	Chlorogenic acid	24,358	3969202	0,82	70168	97,498	ug/ml
26	Pa 280 nm		25,503	3619857	0,75	51833		
27	Pa 280 nm	Vanillic acid	27,549	1673678	0,35	28939	54,279	ug/ml
28	Pa 280 nm	Caffeic acid	28,634	3970818	0,82	34041	44,937	ug/ml
29	Pa 280 nm		31,800	38468	0,01	3111		
30	Pa 280 nm		32,378	23993	0,00	1364		
31	Pa 280 nm	Syringic acid	33,287	104979	0,02	3054	3,826	ug/ml
32	Pa 280 nm		34,333	13022	0,00	752		
33	Pa 280 nm		35,433	20709	0,00	1789		
34	Pa 280 nm		35,858	217093	0,04	4952		
35	Pa 280 nm	(-)-Epicatechin	36,692	88095	0,02	3218	13,061	ug/ml
36	Pa 280 nm		37,228	85647	0,02	3113		
37	Pa 280 nm		37,681	126178	0,03	3487		
38	Pa 280 nm		41,245	2805060	0,58	39737		
39	Pa 280 nm		41,777	993289	0,21	38693		
40	Pa 280 nm	p-Coumaric acid	42,552	2094282	0,43	48700	14,457	ug/ml
41	Pa 280 nm		42,979	970892	0,20	45073		
42	Pa 280 nm		44,136	14374593	2,98	411327		
43	Pa 280 nm		44,705	11950797	2,48	318959		
44	Pa 280 nm	Ferulic acid	45,672	4292930	0,89	116920	53,845	ug/ml
45	Pa 280 nm		46,248	751758	0,16	68769		
46	Pa 280 nm		46,437	943566	0,20	67929		

	Channel Description	Peak Name	RT (min)	Area (V*sec)	% Area	Height (V)	Amount	Units
47	Pa 280 nm		47,031	2455902	0,51	81459		
48	Pa 280 nm		47,414	2809484	0,58	88107		
49	Pa 280 nm		47,833	1253615	0,26	84570		
50	Pa 280 nm		48,275	3603398	0,75	136014		
51	Pa 280 nm		48,909	9093133	1,88	333058		
52	Pa 280 nm		49,305	5270252	1,09	288314		
53	Pa 280 nm	Salicylic acid	49,753	36230142	7,51	1719220	2254,173	ug/ml
54	Pa 280 nm		50,177	70293270	14,56	1777517		
55	Pa 280 nm		51,083	22558761	4,67	537269		
56	Pa 280 nm		52,246	12424159	2,57	214945		
57	Pa 280 nm	Rutin	53,446	5583372	1,16	165373	343,925	ug/ml
58	Pa 280 nm	Hesperidin	54,254	15372196	3,19	208498	250,890	ug/ml
59	Pa 280 nm		55,716	3472592	0,72	135219		
60	Pa 280 nm	Rosmarinic acid	55,914	1599517	0,33	134130	33,941	ug/ml
61	Pa 280 nm		56,643	16390574	3,40	271373		
62	Pa 280 nm		57,560	28393206	5,88	239333		
63	Pa 280 nm		60,700	511958	0,11	74180		
64	Pa 280 nm		61,377	7555607	1,57	241568		
65	Pa 280 nm		62,351	29215817	6,05	519224		
66	Pa 280 nm		62,972	5052231	1,05	406316		
67	Pa 280 nm		63,140	6437541	1,33	374288		
68	Pa 280 nm	Quercetin	63,525	3825533	0,79	338662	121,078	ug/ml
69	Pa 280 nm		63,716	5014718	1,04	354264		
70	Pa 280 nm		63,970	4223756	0,88	377134		
71	Pa 280 nm		64,308	10629811	2,20	794371		
72	Pa 280 nm		64,476	28971565	6,00	975182		
73	Pa 280 nm		65,411	4151548	0,86	237236		
74	Pa 280 nm	Kaempherol	65,685	2969778	0,62	196178	86,740	ug/ml
75	Pa 280 nm		66,050	4406785	0,91	258818		
76	Pa 280 nm		66,326	9471568	1,96	237651		
77	Pa 280 nm		67,409	1976460	0,41	117083		
78	Pa 280 nm		67,687	2880579	0,60	124803		
79	Pa 280 nm		68,083	2407028	0,50	97100		

	Channel Description	Peak Name	RT (min)	Area (V*sec)	% Area	Height (V)	Amount	Units
80	Pa 280 nm		68,587	1446731	0,30	89030		
81	Pa 280 nm		68,833	4071870	0,84	90480		
82	Pa 280 nm		69,737	1891156	0,39	127068		
83	Pa 280 nm		69,950	6663891	1,38	143497		
84	Pa 280 nm		71,036	1133333	0,23	67264		
85	Pa 280 nm		71,377	1781558	0,37	68110		
86	Pa 280 nm		72,088	2150712	0,45	68962		
87	Pa 280 nm		72,428	1919367	0,40	81036		
88	Pa 280 nm		72,823	4873308	1,01	88141		
89	Pa 280 nm		73,771	3898493	0,81	74943		

IM-BAS

Project Name: project

Reported by User: System

SAMPLE INFORMATION

Sample Name:	BBF_1	Acquired By:	System
Sample Type:	Unknown	Sample Set Name:	
Vial:	999	Acq. Method:	PolyPhenols
Injection #:	4	Date Acquired:	02.9.2021 a. 13:13:13
Run Time:	75,00 Minutes	Injection Volume:	20,00 ul

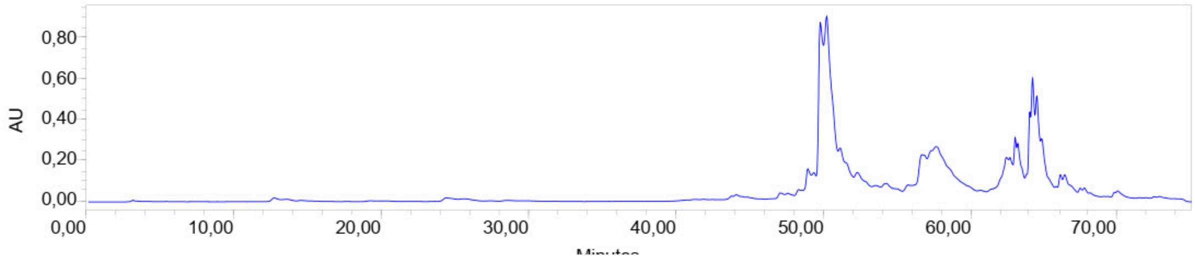

Channel: 2487Channel 2 Channel Desc.: FL 360 nm Processing Method: PolyPhenols UV

	Channel Description	Peak Name	RT (min)	Area (V*sec)	% Area	Height (V)	Amount	Units
1	FL 360 nm		3,079	122367	0,06	7676		
2	FL 360 nm		3,300	52822	0,02	3324		
3	FL 360 nm		9,789	10130	0,00	320		
4	FL 360 nm		11,762	19832	0,01	399		
5	FL 360 nm		12,671	610790	0,28	17851		
6	FL 360 nm		13,495	529277	0,24	11459		
7	FL 360 nm		14,465	373393	0,17	5603		
8	FL 360 nm		17,587	12113	0,01	415		
9	FL 360 nm		19,181	166254	0,08	3929		
10	FL 360 nm		20,094	163064	0,07	3147		
11	FL 360 nm		23,581	21965	0,01	874		
12	FL 360 nm	Chlorogenic acid	24,358	941464	0,43	18209	69,620	ug/ml
13	FL 360 nm		25,540	904860	0,42	13374		

	Channel Description	Peak Name	RT (min)	Area (V*sec)	% Area	Height (V)	Amount	Units
14	FL 360 nm		27,299	75068	0,03	3025		
15	FL 360 nm		27,412	74105	0,03	2988		
16	FL 360 nm	Caffeic acid	28,461	291331	0,13	4956	15,116	ug/ml
17	FL 360 nm		29,736	204124	0,09	3247		
18	FL 360 nm		41,143	227836	0,10	4431		
19	FL 360 nm		41,367	45467	0,02	3956		
20	FL 360 nm		41,791	99376	0,05	3741		
21	FL 360 nm		42,420	22168	0,01	1318		
22	FL 360 nm		43,721	315208	0,14	18171		
23	FL 360 nm		44,045	912326	0,42	24346		
24	FL 360 nm	Ferulic acid	44,782	624676	0,29	13511	43,732	ug/ml
25	FL 360 nm		46,508	173774	0,08	5901		
26	FL 360 nm		47,038	981973	0,45	34337		
27	FL 360 nm		47,552	1088922	0,50	32917		
28	FL 360 nm		48,285	1281980	0,59	51029		
29	FL 360 nm		48,908	3788340	1,74	153957		
30	FL 360 nm		49,314	2552252	1,17	134535		
31	FL 360 nm		49,754	18069402	8,30	872571		
32	FL 360 nm		50,178	34632353	15,92	903122		
33	FL 360 nm		51,093	11821359	5,43	256599		
34	FL 360 nm		52,258	7602715	3,49	136658		
35	FL 360 nm	Rutin	53,451	2525219	1,16	72901	66,689	ug/ml
36	FL 360 nm		54,235	4058832	1,87	83586		
37	FL 360 nm		54,800	1701504	0,78	58307		
38	FL 360 nm		55,711	2086815	0,96	76936		
39	FL 360 nm	Rosmarinic acid	55,939	668475	0,31	74466	38,073	ug/ml
40	FL 360 nm		56,656	9354948	4,30	225878		
41	FL 360 nm		57,618	30639833	14,08	265300		
42	FL 360 nm		60,654	1822211	0,84	52187		
43	FL 360 nm		62,388	9309827	4,28	213686		
44	FL 360 nm		62,632	3579892	1,65	213786		
45	FL 360 nm		62,989	3822067	1,76	311547		
46	FL 360 nm	Quercetin	63,164	6427395	2,95	282954	74,073	ug/ml

IM-BAS

Project Name: project
Reported by User: System

	Channel Description	Peak Name	RT (min)	Area (V*sec)	% Area	Height (V)	Amount	Units
47	FL 360 nm		63,984	5510740	2,53	436817		
48	FL 360 nm		64,170	9063171	4,17	602847		
49	FL 360 nm		64,456	9237324	4,25	516780		
50	FL 360 nm		64,786	9077992	4,17	306594		
51	FL 360 nm	Kaempherol	65,767	843082	0,39	72177	6,851	ug/ml
52	FL 360 nm		66,058	2216748	1,02	130126		
53	FL 360 nm		66,351	5112018	2,35	129970		
54	FL 360 nm		67,402	1129254	0,52	64294		
55	FL 360 nm		67,692	1411792	0,65	65434		
56	FL 360 nm		68,072	1541309	0,71	41801		
57	FL 360 nm		69,015	478951	0,22	24753		
58	FL 360 nm		69,269	477272	0,22	24860		
59	FL 360 nm		69,743	656790	0,30	44636		
60	FL 360 nm		69,949	2374824	1,09	51846		
61	FL 360 nm		71,414	560951	0,26	19429		
62	FL 360 nm		71,844	542188	0,25	19219		
63	FL 360 nm		72,421	593563	0,27	24199		
64	FL 360 nm		72,791	1181715	0,54	25779		
65	FL 360 nm		73,758	757004	0,35	14946		
